# Anti-proliferative effect of Cannabidiol in Prostate cancer cell PC3 is mediated by apoptotic cell death, NFκB activation, increased oxidative stress, and lower reduced glutathione status

**DOI:** 10.1371/journal.pone.0286758

**Published:** 2023-10-05

**Authors:** Jie Li, Tengfei Gu, Shengping Hu, Baiye Jin

**Affiliations:** 1 Department of Urology, Zhejiang University School of Medicine First Affiliated Hospital, Hangzhou, China; 2 Department of Urology, Lishui Central Hospital and Fifth Affiliated Hospital of Wenzhou Medical College, Lishui, China; 3 Zhejiang Engineering Research Center for Urinary Bladder Carcinoma Innovation Diagnosis and Treatment, Hangzhou, China; University of Catania, ITALY

## Abstract

Prostate cancer is the second most frequent cancer diagnosed in men in the world today. Almost all prostate cancers are adenocarcinomas and develop from gland cells. We used the PC3 prostate cancer cell line, which is well studied and derived from a bone metastasis of a grade IV prostatic adenocarcinoma. Cannabidiol (CBD), a major non-psychoactive constituent of cannabis, is a cannabinoid with anti-tumor properties but its effects on prostate cancer cells are not studied in detail. Here, we found cannabidiol decreased prostate cancer cell (PC3) viability up to 37.25% and induced apoptotic cell death in a time and dose-dependent manner. We found that CBD activated the caspases 3/7 pathways and increased DNA fragmentation. Furthermore, we observed an increase of pro-apoptotic genes Bax, an increased level of reactive oxygen species, lower reduced glutathione level, and altered mitochondrial potential in response to CBD treatment leading to lower cellular ATP. Overall, our results suggest that CBD may be effective against prostate cancer cells.

## Introduction

Prostate cancer (PC) is the most commonly diagnosed non-skin cancer and the second leading cause of cancer deaths in men in the United States (CDC Prostate Cancer Statistics 2022). Early tumors are androgen-dependent and can be controlled by surgical procedures, but it relapses with devasting result in morbidity and mortality rates [1]. Prostate cancer risk among men is strongly associated with a family history [2]. The prognosis for men with prostate cancer is dependent on tumor grade at primary diagnosis, which is mainly PSA testing and digital rectal examination [3]. Metastatic prostate cancer, advanced disease states, is no longer organ-confined and involves mainly lymph nodes and/or bone. There is currently no curative treatment against this type of prostate cancer and targeting prostate cancer cells may represent a better therapeutic strategy for the treatment.

Although most prostate cancer (>90%) is organ-confined or locally advanced, treatment options were for active surveillance, local radiotherapy or prostatectomy [4,5]. Androgen deprivation therapy (ADT) by surgical or chemical castration to decrease circulating testosterone levels is often used as primary therapy [6]. In addition multiple other therapy such as compounds targeting androgen signaling, signaling pathway inhibitors, DNA damage repair pathway modulators, prostate-specific membrane antigen (PSMA) targeting and immunotherapy [7]. Despite these advances, there is strong need for additional therapy development.

NF-κB transcription factors regulate the expression of hundreds of genes in cancer cells that are involved in regulating cell growth, differentiation, development, and apoptosis [8]. Reactive oxygen species(ROS) have critical role in cellular death in cancer cells and involved in the crosstalk with NF-κB [9]. ROS can trigger both apoptotic and necrotic cell death depending its levels [10] and NF-κB plays important role. Targets of NF-κB include gp91phox, and iNOS [11,12]. NADPH oxidase (gp91phox) produces superoxide whereas iNOS produce nitric oxide. These two ROS can generate peroxynitrite, a fast killing ROS. Cyp2E1, also regulated by NF-κB, can produce ROS whereas Gpx1, also regulated by NF-κB, is an anti-oxidant gene and maintain the balance of ROS in cells[13,14].

The use of cannabinoids containing plant ext racts as herbal medicine can be traced back to as early as 500 BC in Asia and Chinese traditional medicine. Recently antiproliferative, proapoptotic and proautophagic effects of cannabinoids on cancer cells has been reported [15]. Both CB1 and CB2 are seven-transmembrane domain receptors coupled to Gi/o protein and its activation triggers signalling pathways widely involved in cancer [16,17]. Recently, non-psychotic cannabinoids, cannabidiol or CBD, have garnered tremendous attention for health-related uses and are promising anti-cancer drugs [18,19]. It was observed that CBD can act as an antagonist to the CB1 receptor in vitro [20]. Although, plant-derived extracts containing CBD show anti-cancer activity on prostate cell lines, little is known about the mechanism. CBD also inhibits tumor angiogenesis [21]. In this study, we systematically demonstrated the cell death mechanism of CBD in the PC3 prostate cancer cell line through apoptotic pathways and involvement of NFkB/mitochondria/cellular redox status.

## Method

### Cell culture

PC3, a prostate cancer cell line, was obtained from ATCC and cultured in RPMI-1640 medium (Sigma-Aldrich) supplemented with 10% fetal bovine serum (Invitrogen; Thermo Fisher Scientific, Inc.), at 37°C and 5% CO2. Cells were serum deprived before treatment with CBD.

### Drug

Cannabidiol(CBD) was purchased from Push (Chengdu, China) and dissolved in 50mM DMSO stock, and prepared fresh each day.

### Cell viability by XTT assay

PC3 cell survival was determined by XTT Assay Kit (Abcam) according to manufacturer instructions. Briefly, cells were seeded in 96 well plate and allowed to grow at 80% confluence. Cells were treated with CBD at the concentration/time described with each experiments. XTT reagents were added 4 hour before reading in spectrophotometer.

### DNA fragmentation

Cellular DNA Fragmentation of PC3 cells with or without treatments were analyzed with In Situ Cell Death Detection Kit (Roche, Millipore -Sigma) and followed the manufacturer’s instruction.

### Homogeneous Caspase-3/7 Assay

Apoptosis assays were performed from PC3 cell lysates with the Promega Apo-ONE Homogeneous Caspase-3/7 Assay kit according to manufacturer instructions.

### RNA isolation

Total RNAs were isolated by the standard trizol method with a Qiagen RNA isolation kit. Briefly, 1X107 cells were harvested and processed with Trizol according to manufacterers instructions.

### Real-Time qPCR

Total RNA was extracted from PC3 cells using a Qiagen RNA Kit I (Qiagen, China) and the mRNAs were reversely transcribed into cDNA with a cDNA kit reagent Kit (Takara Bio, Inc., Dalian, China). RT-qPCR analysis was performed by using the SYBR Premix Ex Taq II kit (TaKaRa). The thermocycling conditions included: 95˚C for 1 min, followed by 40 cycles of amplification at 95˚C for 5 sec and 60˚C for 30 sec. The levels of transcripts were expressed as the comparative Ct method. All primers were obtained from Qiagen(China).

### Caspase 3/7 detection in live cells

CellEvent Caspase-3/7 Green detection reagents (Thermo Fisher Scientific) are used as fluorogenic substrates for activated caspase-3/7 in live cells along with live cell nuclear stain Hoechst 33342 Solution (Thermo Fisher Scientific). Fluorescent imaging was performed with 40X objectives.

### Intracellular staining of NFKB

PC3 cells were grown in glass bottom dishes and fixed with 4% paraformaldehyde after desired treatments. After cell permeabilization and blocking, cells were stained with NFkB antibody (1:100 dilution) overnight. After washing, the glass bottom dishes with stained cells were incubated with Alexa Fluor 547 secondary antibody followed by wash and DAPI staining. Fluorescent imaging was performed with 40X objectives.

### Intracellular ROS

The intracellular ROS in prostate cancer cells PC3, an assay kit (Beyotime Institute of Biotechnology) was used. Briefly, following pretreatment for 48 h cells were collected and resuspended in PBS containing dichlorofluorescein diacetate. Following incubation at 37ªC for 10 min the cells were harvested and analyzed using flow cytometry.

### Mitochondrial membrane potential

PC3 cells were plated and cultured for 24 h, then exposed to CBD as described concentration for an additional 48h. The JC-1 mitochondrial membrane potential assay kit (Beyotime Institute of Biotechnology) was used and analyzed using flow cytometry.

### Reduced /Oxidized Glutathione assay

Cells were plated and exposed to CBD for an additional 48h at 10μM. The cells were lysed, and the supernatant was collected by centrifuging at 10,000 x g at 4ªC for 10 min and then determined for reduced glutathione (GSH) and oxidized glutathione (GSSG) levels using GSH and GSSG assay kit (Beyotime Institute of Biotechnology) according to the manufacturer’s instruction.

### Intracellular adenosine triphosphate (ATP) levels

Intracellular ATP levels were measured using an ATP assay kit (Abcam) according to the manufacturer’s instructions. Briefly, PC3 cells were processed, and the supernatant of the lysis buffer was then collected by centrifugation at 10,000 x g at 4C for 12 min. Colorimetric assays were performed in a plate reader.

### Apoptotic cell death by flow cytometry

Set were incubated with Annexin V FITC and PI using Annexin V-FITC Apoptosis Detection Kit (Sigma, China). The AnnexinV-FITC kit uses annexin V conjugated with fluorescein isothiocyante (FITC) to label apoptotic cells. The kit also includes propidium iodide (PI) to label the cellular DNA in necrotic cells. This combination allows the differentiation among early apoptotic cells (annexin V positive, PI negative), necrotic cells (annexin V positive, PI positive), and viable cells (annexin V negative, PI negative).

### Western blot analyses

Western blot was performed as described previously [22].

### Statistical analysis

All experiments were performed in two sets of triplicates. The results are presented as the mean ± standard error of the mean. The statistical analyses were performed in Excel and the statistical notation as described in the figure legends. Students t-tests were performed with nonparametric unpaired test were performed with two tailed distribution.

## Results

### CBD reduced PC3 prostate cancer cells survival in a dose-dependent manner and induced apoptotic pathways

**Fig 1:**
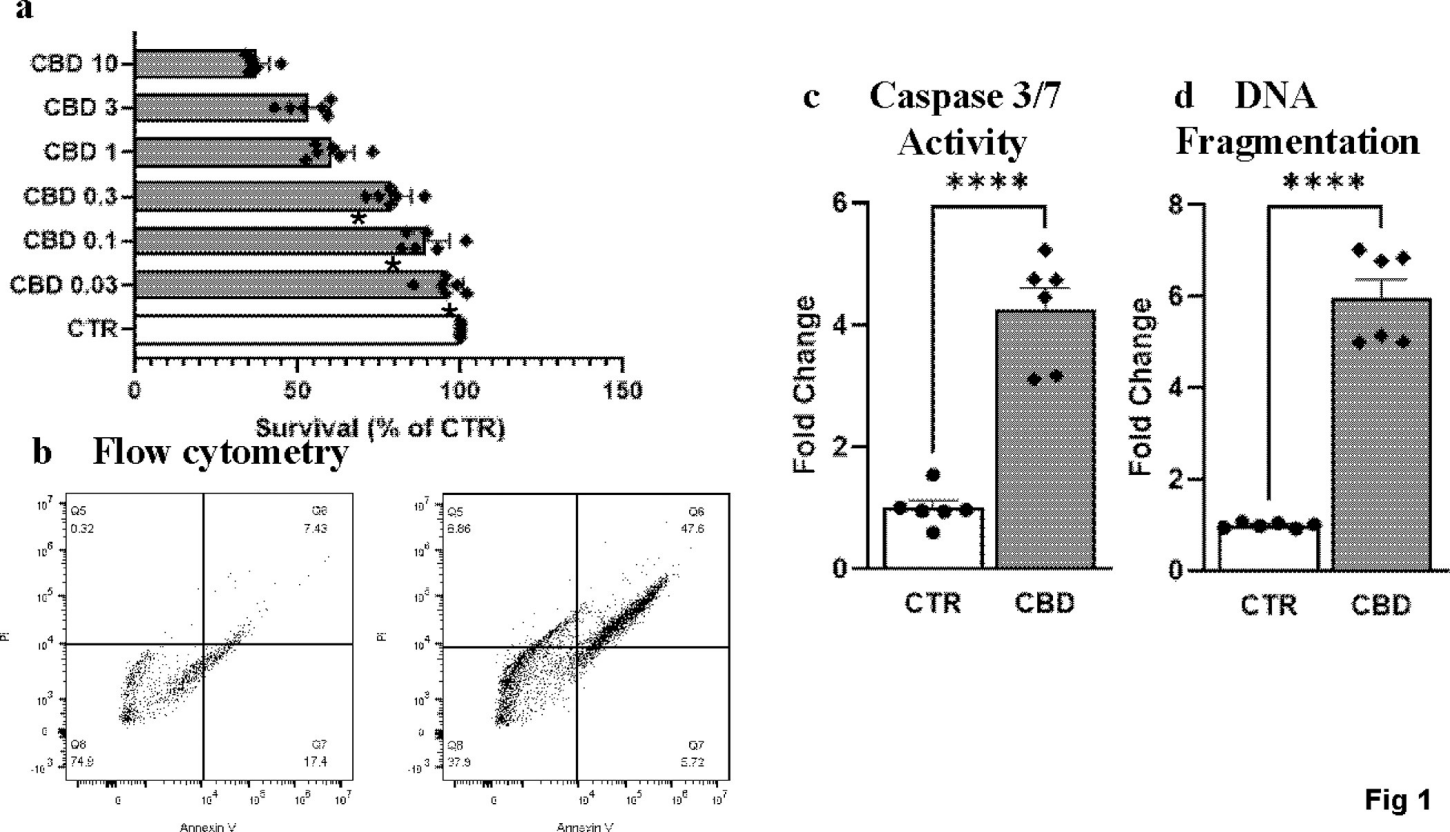
Effect of CBD in PC3 prostate cancer cells survival and apoptotic pathways. (a) XTT assay demonstrated CBD (0.03–10 μM) dose-dependently reduced cell survivals at 48h. A significant difference was observed at 1, 3, and 10 μM doses only. (b) Flow cytometric analyses of PC3 cells treated with 10μM CBD. Significant increase in (c) caspase 3/7 activity and (d) DNA fragmentation after CBD treatment (10μM) for 48h. **** p<0.0001 CBD vs CTR, n = 6/group.

We examined the dose-dependent effect of CBD in the PC3 cell line. To assess the survival rate PC3 cells were treated with a range of CBD concentrations for 48h and XTT assays were performed. CBD treatments for 48h lead to survivals (%) of 89.46±2.97, 78.66±2.512, 60.09±3.012, 53.15± 2.849, 37.25±1.599 at 0.1, 0.3, 1, 3 and 10 μM respectively (Fig 1A). Flowcytometry analyse with annexin V and PI with control and CBD at 10 μM demonstrated similar pattern (Fig 1B). To assess whether CBD induced apoptosis in PC3 cells, we determined two apoptosis markers caspase 3/7 activity and DNA fragmentation. CBD treatment at 10 μM for 48h led to a 4.24±0.36-fold increase in caspase 3/7 activity and a 5.96±0.41 fold increase in DNA fragmentation (Fig 1C and 1D). These data confirmed the induction of apoptosis in PC3 cells by CBD.

### The CBD-induced intrinsic pathway of apoptosis in PC3 cells

**Fig 2:**
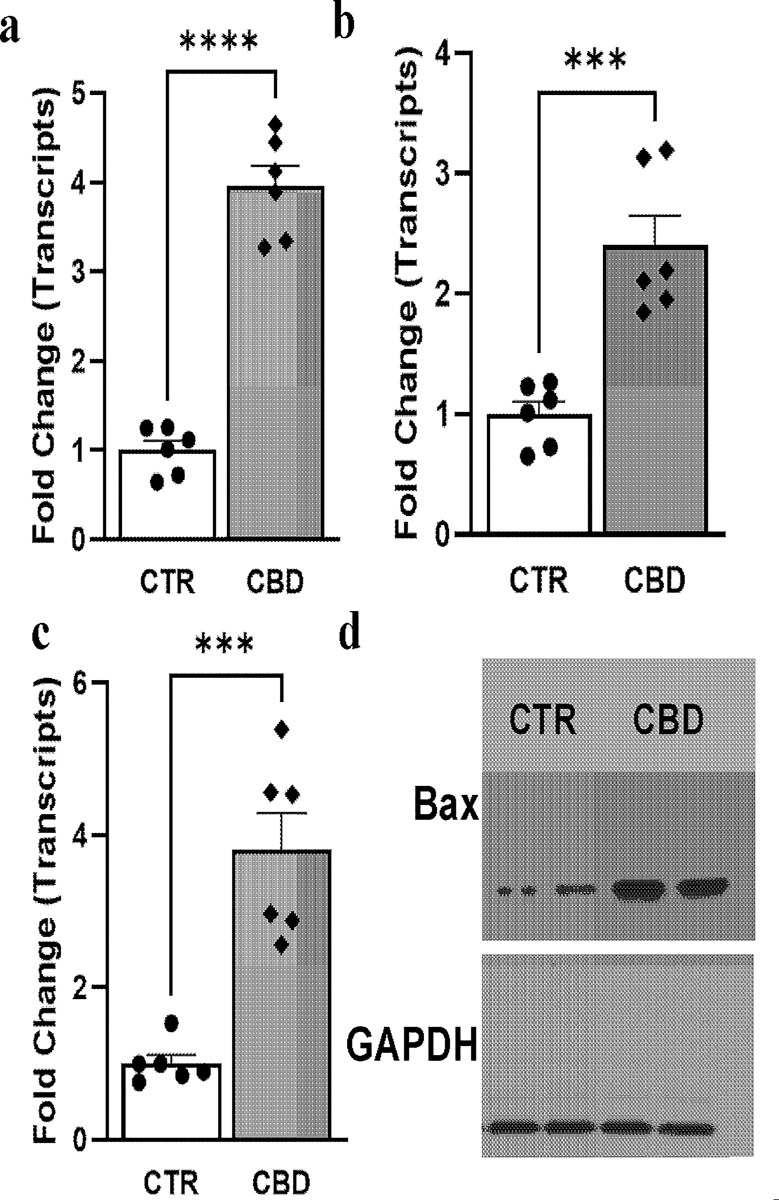
Effect of CBD on the intrinsic pathway of apoptosis in PC3 cells. Real-time PCR analyses of transcripts (a) Caspase 3 (b) Caspase 9 and (c) Bax from PC3 cells treated with vehicle (CTR) or CBD (10μM) for 48h. All data were normalized to the housekeeping gene β-actin. *** p<0.001 CBD vs CTR; **** p<0.0001 CBD vs CTR, n = 6/group. (d) Western blot analyses of Bax proteins from control and CBD treated PC3 cells.

**Fig 3:**
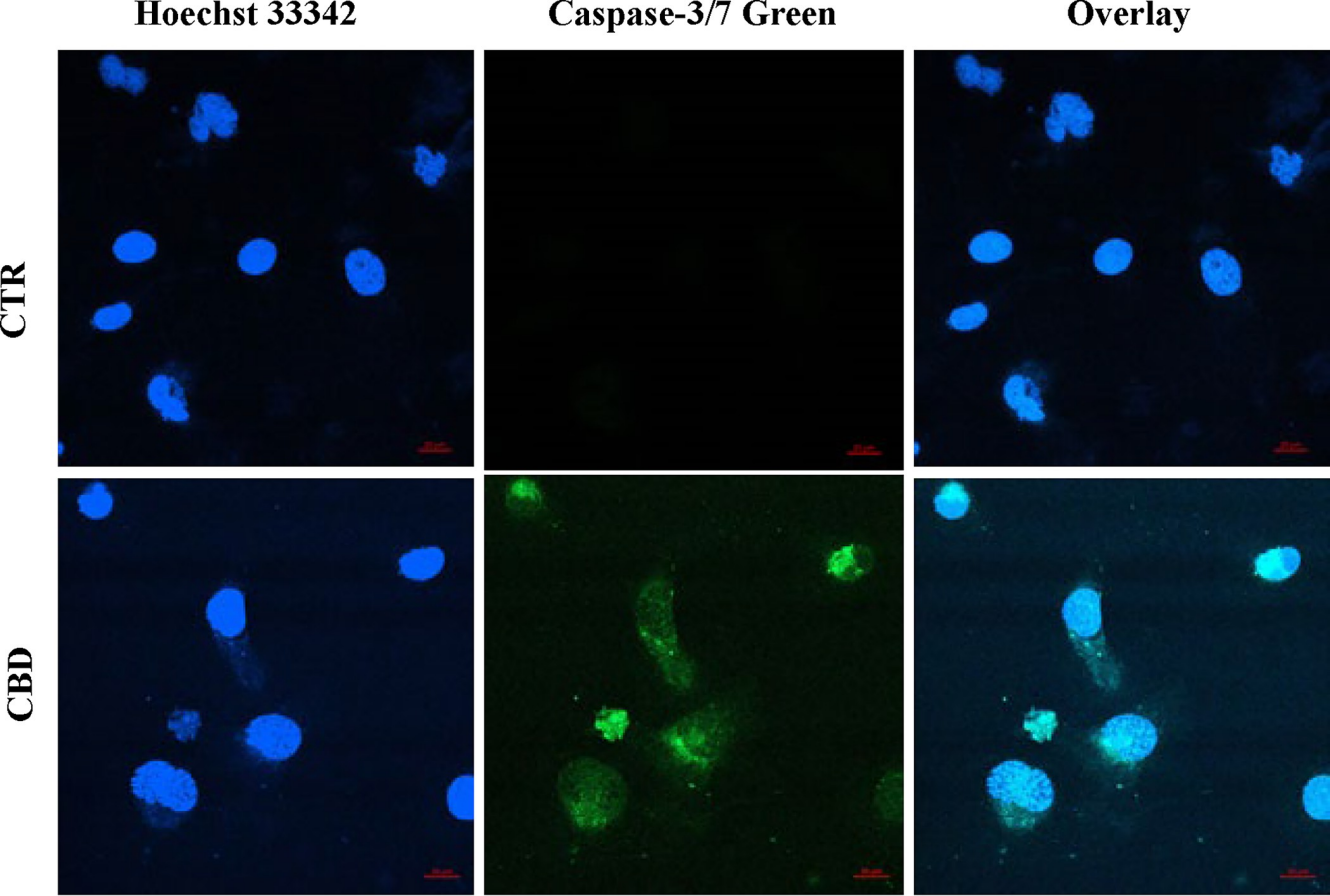
Effect of CBD on caspase 3/7 activation in PC3 cells. CBD treatment (10μM) for 48h led to the induction of caspase 3/7 activation (green color) of apoptotic pathways in most cells as determined by fluorescence imaging along with nuclear stain(blue). Scale bars were provided on the right side bottom of all images. A few cells also demonstrated apoptotic nuclei (fragmented blue).

To examine further whether intrinsic pathways of apoptosis were involved in CBD-induced cell death, real-time PCR analyses were performed from isolated RNA. Real-time PCR analyses of transcripts demonstrated an increase in all common intrinsic pathways genes namely Caspase 3, Caspase 9, Bax, and Bcl-2 in PC3 cells treated with vehicle (CTR) or CBD (10μM) for 48h (Fig 2A–2D). Quantitative determination by comparative Ct method of real-time PCR showed a 3.95±0.23 fold increase in Caspase 3, 2.4±0.24 fold increase in Caspase 9, 3.81±0.47 fold increase in Bax and similar pattern increase of Bax expression by western blot analyses. All these real-time PCR data suggested the onset of intrinsic pathways of apoptosis by CBD. To verify further at the activity level, caspase 3/7 green was added to the cells and CBD treatment at 10 μM for 48h led to induction of caspase 3/7 substrate usage as shown by the green color in most cells (Fig 3) whereas no substrate usage was observed in the vehicle-treated group (CTR).

### CBD-induced NFKB regulation and downstream effector transcript for cell death pathways in PC3 cells

**Fig 4:**
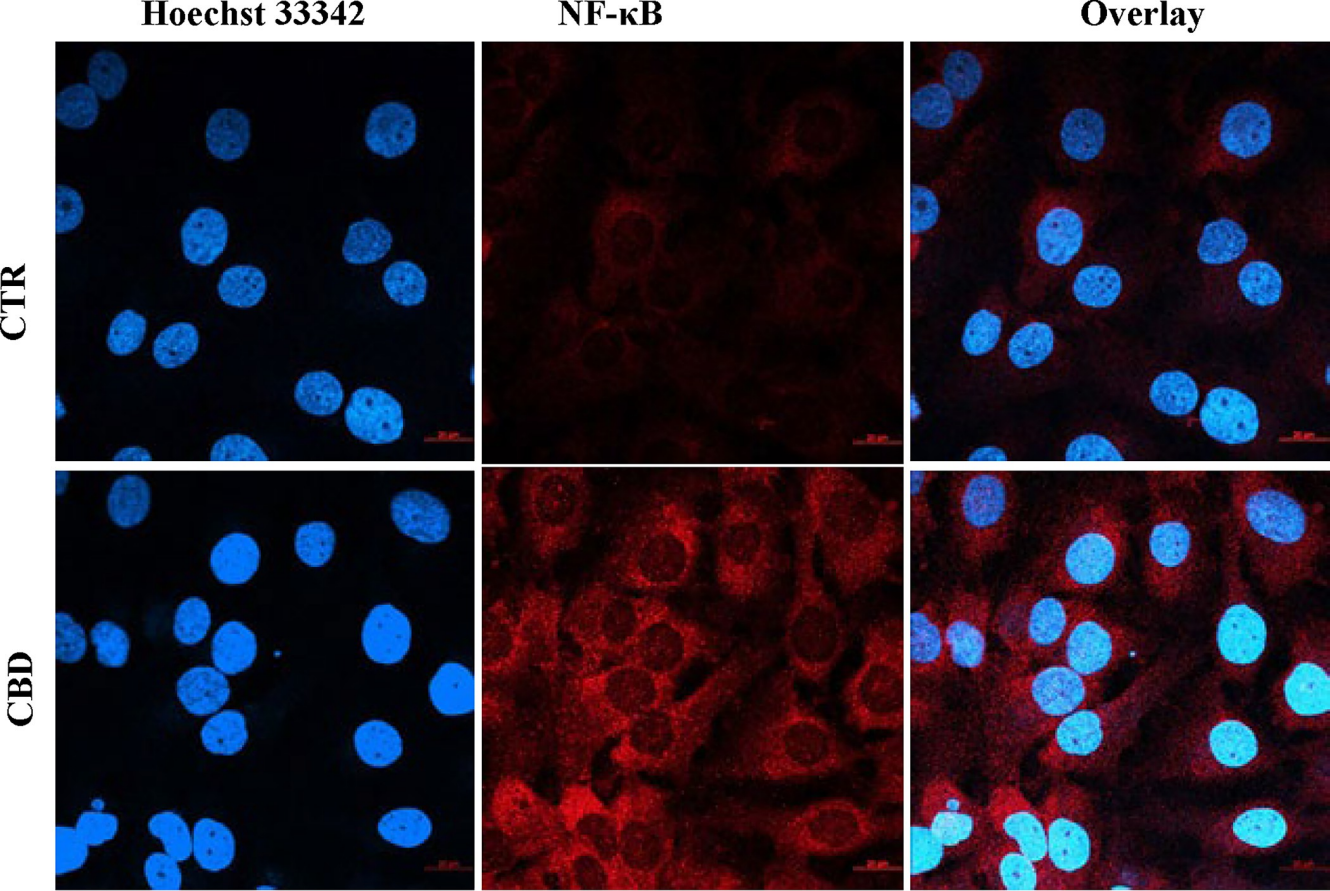
Effect of CBD in NFKB regulation in PC3 cells. CBD treatment (10μM) for 48h led to induction of NFkB (red colour) in most cells and its nuclear localization (overlapped with blue nuclear stain). Scale bars were provided on the right side bottom of all images.

**Fig 5:**
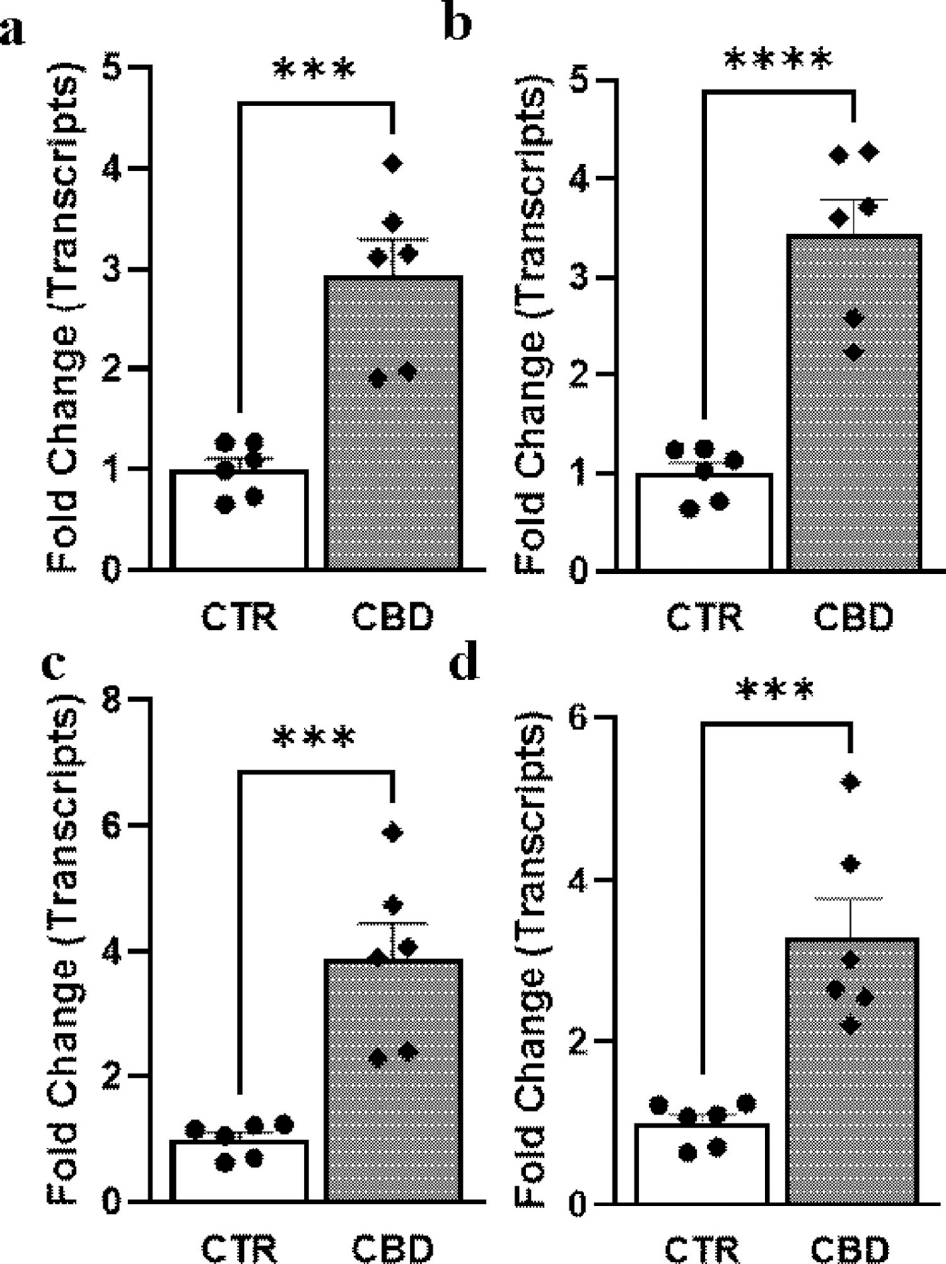
Effect of CBD in NFKB downstream effector transcripts for non-canonical cell death pathways. Real-time PCR analyses of transcripts (a) gp91phox (b) iNOS (c) Cyp2E1 and (d) Gpx1 from PC3 cells treated with vehicle (CTR) or CBD (10μM). All data were normalized to the housekeeping gene β-actin. *** p<0.001 CBD vs CTR; **** p<0.0001 CBD vs CTR, n = 6/group.

CBD treatment at 10 μM for 48h led to the induction of p65 NFkB and its nuclear localisations shown by intracellular staining and fluorescence microscopy (Fig 4). Most cells were shown to be positive cellular and nuclear staining in the CBD treatment group whereas only a few cells show positive in the CTR group. CBD has been shown to have induced NFkb in glioblastoma cells earlier [23]. We examined further to understand whether its downstream effector genes were upregulated. Real-time PCR analyses of gp91phox, iNOS, Cyp2E1 and Gpx1 transcripts were increased with CBD (10μM) at 2.94±0.34, 3.44±0.35, 3.89±0.56 and 3.305±0.47 fold respectively (Fig 5A–5D). These genes are suggestive of increased oxidative stress in response to CBD.

### CBD increases intracellular ROS, decreased cellular reduced glutathione levels, and decreased mitochondrial potential and ATP content in PC3 cells

**Fig 6:**
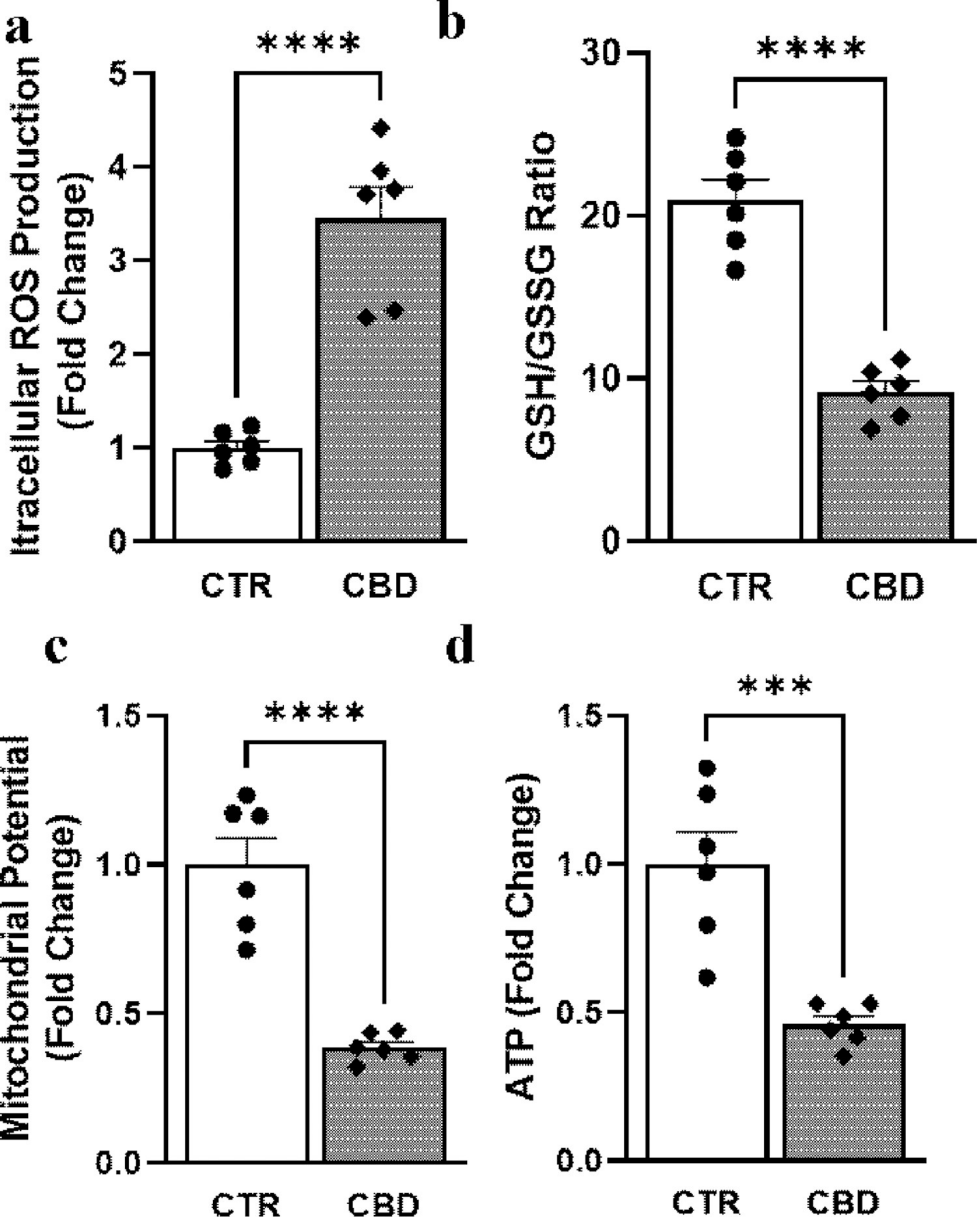
Effect of CBD on intracellular ROS, cellular glutathione balance, mitochondrial potential, and ATP content in PC3 cells. Quantitative measurements of (a) intracellular ROS (b) reduced and oxidized glutathione ratio (c) Mitochondrial membrane potential and (d) ATP generation from PC3 cells treated with vehicle (CTR) or CBD (10μM). Statistical analyses: *** p<0.001 CBD vs CTR; **** p<0.0001 CBD vs CTR, n = 6/group.

We further investigated the CBD effect of NFkB activation in PC3 cells by looking at downstream endpoints. Intracellular measurement of ROS showed a 3.45±0.34 fold increase with CBD treatment (Fig 6A). Reduced glutathione reserve is a key element to cellular redox status and the ratio decreased by 50% in response to CBD treatment (Fig 6B). We also measured mitochondrial potential, which also dropped to 38.7% in response to CBD treatment compared to the control group. ATP, a key molecule of cellular energy status also decreased to 0.45 fold (Fig 6C and 6D).

## Discussion

Cannabinoids have received special attention in recent years due to their unique pharmacologic properties including anti-inflammatory, cell cycle modulation, and anti-tumor properties. Cannabinoid groups include a wide array of molecules, few are physiologically produced in humans, called endocannabinoids. Endocannabinoids interacts with cells through two cannabinoid receptors CB1 an CB2 [24]. CB1 receptors mainly abundants in neural cells whereas Cb2 receptor is mainly in immune cells. However, CB1 receptor in heart, liver and other organis plays major role in many pathological condition[25,26]. CB1 receptor is primary focus in recent year for its major role in cance r[16,27,28].

Many cannabinoids are synthesized in laboratories, and many are extracted primarily from the Cannabis sativa plant. Among plant-derived cannabinoids, CBD does not have any psychotic effect and is established as an anti-inflammatory agent. In recent years, the anti-cancer effect of CBD on lung cancer, breast cancer, Leukemia/Lymphoma, and glioma gave a tremendous interest to understand its role in therapeutic aspects [29–33]. Anti-proliferative effect of CBD in some prostate cancer cells is reported but the mechanism at the molecular level is not well understood [34]. In this study, we demonstrated apoptotic pathway is activated via an intrinsic pathway in prostate cancer cells PC3. We also observed induction and nuclear localization of p65 NFkB in PC3 cells and induction of downstream targets namely gp91 phox, iNOS, Cyp2E1, and Gpx1. The discover of NADPH oxidases help to understand how reactive oxygen species were generated in cancer cells [35]. Gp91 phox, one of the major subunit of NADPH oxidases, playes critical role in ROS generation in prostate cancer [36,37]. iNOS-derived NO and its tumorigenic or tumoricidal activitieshave been reported earlier [38]. CYP2E1 has been associated with altered susceptibility with increased risk of development of other malignant tumors such as lung cancer [39,40]. Increase in those gene by CBD have direct implicatuin in cancer cell survival. Gpx1, antioxidant gene also induced by CBD and have role in cancer [41]. Further examination led to conclude CBD increased oxidative stress in cancer cells, reduced glutathione reserve, and reduced mitochondrial potential, which lead to reduced ATP status in PC3 cells.

First, we observed that CBD reduced prostate cancer cell survival in a dose-dependent manner and induced apoptotic pathways by inducing caspase 3/7 activity and DNA fragmentation. Our findings are consistent with earlier publications stating the anti-proliferative and pro-apoptotic effects of CBD [18]. Cannabidiol inhibits the viability of breast cancer cells by increasing DNA fragmentation and inducing apoptotic pathways[42]. CBD also induces apoptosis in breast cancer cells and colorectal cancer cells [43,44]. To understand further, we observed the induction of mitochondrial genes Bax, and thus involving intrinsic pathways of apoptosis. Shrivastava et al also demonstrated the involvement of intrinsic pathways in breast cancer cells and cannabidiol has a direct effect on mitochondria [43,45].

The upregulation and nuclear localization of p65 NFkB in PC3 cells are our critical findings. To establish further, we demonstrated its downstream effector genes, which were also induced and have a direct role in apoptosis. It has been shown earlier that CBD induction of NFkB mediated anti-inflammatory and other downstream pathways [46–48]. We have observed the induction of non-canonical pathways of NFkB activation, which lead induction of oxidative stress.

Finally, we have demonstrated that CBD mediated oxidative stress by increasing intracellular ROS and reducing cellular glutathione levels resulting decrease in mitochondrial potential and ATP production. As shown recently in breast cancer cell and lung cancer cells, CBD induced oxidative stress and caused cell death [49,50]. It is also shown in oral cancer cells that CBD-induced oxidative stress plays a major role in apoptotic cell death [51]. Many scientists are not convinced that how antioxidant compounds like CBD can produce oxidative stress. There are multiple hypotheses to understand this aspect. Primarily, most CBD-induced oxidative stress is reported in cancer cells whereas anti-oxidant properties were reported in normal cells [48,52]. It is important to note that mitochondrial respiration is completely different in two types of cells and thus its response to CBD. Secondly, CBD induces a complex of pathways in cancer cells, which is different in other cell types. Lastly, CBD is shown to induce NRF2 pathways in cancer cells, supporting its role as a pro-oxidant in cancer cell types. However, there are significant research is required to understand fully how CBD mechanistically induced NFR2 [53]. Our study of increased ROS production in PC3 cancer cells will also support that hypothesis.

In summary, we have demonstrated CBD as a potential therapeutic molecule in the treatment of prostate cancer based on its properties of anti-proliferative effect on PC3 cancer cells by promoting intrinsic apoptotic pathway via mitochondrial and NFkB activation followed by intracellular ROS generation and reducing cellular redox status of glutathione.
